# Stilbenoid compounds inhibit NF-κB-mediated inflammatory responses in the *Drosophila* intestine

**DOI:** 10.3389/fimmu.2023.1253805

**Published:** 2023-09-22

**Authors:** Anna L. Aalto, Atefeh Saadabadi, Fanny Lindholm, Christa Kietz, Emmy Himmelroos, Parthiban Marimuthu, Outi M. H. Salo-Ahen, Patrik Eklund, Annika Meinander

**Affiliations:** ^1^ Cell Biology, Faculty of Science and Engineering, Åbo Akademi University, Turku, Finland; ^2^ InFLAMES Research Flagship Center, Åbo Akademi University, Turku, Finland; ^3^ Pharmaceutical Sciences Laboratory, Pharmacy, Åbo Akademi University, Turku, Finland; ^4^ Structural Bioinformatics Laboratory, Biochemistry, Åbo Akademi University, Turku, Finland; ^5^ Laboratory of Molecular Science and Engineering, Faculty of Science and Engineering, Åbo Akademi University, Turku, Finland

**Keywords:** Drosophila, DSS, inflammation, intestine, NF-κB, stilbenoid, TRPA1

## Abstract

**Introduction:**

Stilbenoid compounds have been described to have anti-inflammatory properties in animal models *in vivo*, and have been shown to inhibit Ca2+-influx through the transient receptor potential ankyrin 1 (TrpA1).

**Methods:**

To study how stilbenoid compounds affect inflammatory signaling *in vivo*, we have utilized the fruit fly, *Drosophila melanogaster*, as a model system. To induce intestinal inflammation in the fly, we have fed flies with the intestinal irritant dextran sodium sulphate (DSS).

**Results:**

We found that DSS induces severe changes in the bacteriome of the *Drosophila* intestine, and that this dysbiosis causes activation of the NF-κB transcription factor Relish. We have taken advantage of the DSS-model to study the anti-inflammatory properties of the stilbenoid compounds pinosylvin (PS) and pinosylvin monomethyl ether (PSMME). With the help of *in vivo* approaches, we have identified PS and PSMME to be transient receptor ankyrin 1 (TrpA1)-dependent antagonists of NF-κB-mediated intestinal immune responses in *Drosophila*. We have also computationally predicted the putative antagonist binding sites of these compounds at *Drosophila* TrpA1.

**Discussion:**

Taken together, we show that the stilbenoids PS and PSMME have anti-inflammatory properties *in vivo* in the intestine and can be used to alleviate chemically induced intestinal inflammation in *Drosophila*.

## Introduction

Stilbenoids are hydroxylated derivatives of polyphenolic compounds characterized by a 1,2-diphenylethylnucleus and are present in berries, fruits and grape vine, but also in knots, bark, roots and stumps of conifer trees, such as spruce and pine (1–3). Stilbenoids exhibit antioxidant properties, which protect plants from harmful exogenous stimuli such as excessive heat, UV-light, insect attacks and infections caused by microorganisms (4–6). Some stilbenoid compounds, such as resveratrol (3,4′,5-trihydroxystilbene), pinosylvin (3,5-dihydroxystilbene, PS) and pinosylvin monomethyl ether (3-hydroxy-5-methoxystilbene, PSMME) have been described to have anti-inflammatory properties in animal models *in vivo* (7, 8). In mammalian cells, resveratrol, PS and PSMME have been shown to inhibit Ca^2+^-influx through the transient receptor potential ankyrin 1 (TrpA1) ion channel in response to a potent TrpA1 activator allyl isothiocyanate (AITC) (8, 9).

The ligand-gated, transmembrane (TM) bound TrpA1 receptor is a sensory protein that can be activated by environmental stimuli such as noxious cold and mechanical stimuli, as well as by endogenous irritant and pungent compounds. TrpA1 serves as an attractive target for analgesic and anti-inflammatory drugs, as it is triggered during inflammation, oxidative stress and tissue damage and is considered a key player in acute and chronic pain sensation (10). In flies, TrpA1 is expressed in sensory neurons as well as in the epithelial wall of the intestine (11–14). The receptor has been shown to be involved in oxidative stress-induced intestinal stem cell proliferation and in the clearance of food-borne pathogens in flies (14, 15).

When intestinal homeostasis is disturbed, a local inflammation arises to promote healing and recovery. The *Drosophila* intestinal inflammatory response is induced by epithelial cells that recognise and respond to pathogen-associated molecular patterns (PAMPs). These PAMPs are derived from foreign bacteria or induced by pathological changes of the resident microbiome causing dysbiosis. Recognition of harmful bacteria leads to activation of several inflammation-promoting signalling pathways, including the *Drosophila* nuclear factor κB (NF-κB) pathways. The Immune deficiency (Imd)/Relish pathway is activated upon recognition of bacteria by peptidoglycan recognition proteins (PGRPs) and culminates in the transcriptional activation of the NF-κB transcription factor Relish. In the intestinal epithelial cells of the fly, this is the major NF-κB pathway (16–19). Relish activation leads to the transcription of hundreds of genes, including antimicrobial peptides (AMPs) that upon secretion contribute to intestinal immune responses by fending off intruding pathogens (20–22). Due to the advanced innate immune system of *Drosophila*, as well as several structural and functional similarities between the fly and the mammalian intestine, the fruit fly has emerged as an ethical, inexpensive and fast model to study intestinal inflammatory disease (23).

In this study, we induced intestinal inflammation with the intestinal irritant dextran sodium sulphate (DSS), and analysed the anti-inflammatory properties of stilbenoid compounds *in vivo* in *Drosophila*. We found that while the inflammation caused by DSS is dependent on the commensal microbiome, the elevated immune activation induced by Relish target gene expression can be alleviated by inhibition of the TrpA1 channel by stilbenoid-treatment.

## Results

### Molecular modelling of PS and PSMME interactions with *Drosophila* TrpA1

**Figure 1:**
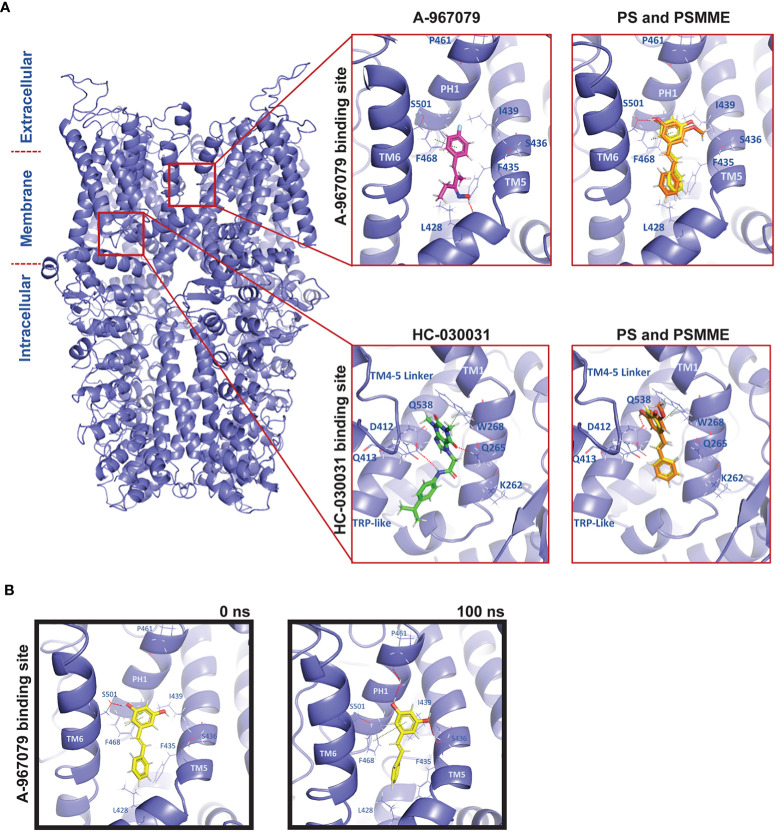
Molecular modelling of PS and PSMME interactions with *Drosophila* TrpA1. **(A)** A comparative homology model of *Drosophila* TrpA1 (blue) based on the experimental human TrpA1 structure (PDB ID: 6V9V). Zoom in: Docking studies of PS (yellow), PSMME (orange) and A-967079 (magenta) or HC-030031 (green) in the A-967079 (top panel) or HC-030031 (lower panel) binding pocket of *Drosophila* TrpA1 (blue). **(B)** PS (yellow) in the A-967079 binding pocket of *Drosophila* TrpA1 (blue), before (left) and after (right) a 100ns MD simulation. The oxygen, nitrogen and hydrogen atoms are displayed in red, blue, and white color, respectively. Interaction color code (dashed lines): H-bond – red; π-π – dark green. Key interacting residues are shown in blue sticks and labelled.

PS and PSMME have been shown to inhibit Ca^2+^-influx through the TrpA1 ion channel in response to TrpA1 activators in mammalian cells (8, 9). The mammalian TrpA1 binding sites have been identified and predicted for two known TrpA1 antagonists, A-967079 and HC-030031, based on phylogenetic, mutational and modelling studies (24). In this study, we have used *Drosophila* as a model to study the effects of stilbenoid compounds on inflammatory responses. To be able to investigate if stilbenes can interact with the *Drosophila* TrpA1 (dTrpA1) channel, we used molecular modelling to computationally predict the antagonist binding sites at the receptor. In the absence of an experimentally defined structure of the dTrpA1 channel, we built a comparative homology model of dTrpA1 based on the experimental human TrpA1 (hTrpA1) structure. The dTrpA1 model with the best Discrete Optimized Protein Energy (DOPE) score (-317763.96875) and the root-mean-square deviation (RMSD) of 0.764 Å from the template was utilized for molecular modelling studies (**Figure 1A**). The two TrpA1 antagonists A-967079 and HC-030031 were used as reference compounds and were modelled into their respective binding sites described in the literature using molecular docking (**Figure 1A**, zoom in). The A-967079 binding site has been described to locate within the channel pore between TM5, TM6 and pore helix 1 (PH1) from one subunit and TM6 from the adjacent chain (25). The residues forming this particular binding pocket in hTrpA1 are S873, T874, F877, F909 and M912 (25–27). The respective residues in dTrpA1 are V431, L432, F435, F468 and M471. The HC-030031 binding pocket is situated between the TM4-TM5 linker, preTM1 and TRP-like domains close to the membrane (28). The residues forming the HC-030031 pocket in hTrpA1 are W711, N855, Q979, H983, A971 and in dTrpA1 W268, Q413, Q538, H542 and A530.

To investigate possible binding modes and sites of stilbenes at dTrpA1, PS and PSMME were docked into both known antagonist binding sites. Based on the docking results, both PS and PSMME are predicted to form a hydrogen bond with S501 (of the adjacent TM6) and π-π interactions with F468 (of PH1) in the A-967079 binding site, while A-967079 itself is predicted to engage in hydrogen bonding with L428 backbone oxygen (in TM5) and π-π interactions with F468 (**Figure 1A**, upper panel). In the HC-030031 binding pocket, PS and PSMME are predicted to form a hydrogen bond with Q538 (of the TRP-like domain) and π-π interactions with W268 (of the preTM1 domain), while HC-030031 itself is predicted to form hydrogen bonds with Q265 (of the preTM1) and D412 (of TM4-TM5 linker) (**Figure 1A**, lower panel).

In addition to assessing the initial docking scores and favorable ligand-protein interactions to evaluate the predicted binding poses, we employed the Molecular Mechanics-Generalized Born Surface Area (MM-GBSA) method to estimate the free energy of binding (binding affinity) of the docked ligands. Moreover, the best docking poses of A-967079 and HC-030031 in their respective dTrpA1 binding sites and PS (as the representative of the stilbene compounds) in both the known binding pockets of the dTrpA1 model were subjected to molecular dynamics (MD) simulation to evaluate the stability of the predicted ligand-dTrpA1 complexes. After the MD simulation, the MM-GBSA binding affinities were recalculated to see if the simulation had improved or worsened the estimated binding affinity of the ligands. Whereas the MM-GBSA binding free energy of HC-030031 was significantly better after the 100-ns simulation (from -44.39 to -74.42 kcal/mol) at its corresponding binding site, it remained the same for A-967079 (-28 kcal/mol), suggesting a less favorable binding for A-967079 at its binding site. The MM-GBSA binding free energies of PS were also improved during the MD simulation (from -35.30 kcal/mol and -31.73 kcal/mol to -45.62 kcal/mol and -54.89 kcal/mol at the A-967079 and HC-030031 binding sites, respectively). PSMME (non-simulated) had very similar binding free energy values to those of PS (non-simulated) at both binding sites.

The predicted HC-030031-TrpA1 complex remained relatively stable during the simulations and HC-030031 engaged in water-mediated hydrogen bonding interactions with Q261 and L264 (of preTM1) (data not shown). However, the small reference ligand A-967079 was not stable in its predicted binding pose at the binding site during the simulation. It changed its position and orientation, and thus, also the interactions with the protein, e.g., the hydrogen bond with L428 (TM5) was exchanged to hydrogen bonds with S501 (of the adjacent TM6), L464 (of PH1) and S436 (of TM5) (data not shown). Although PS binding at the A-967079 site was relatively stable during the whole simulation *via* π-π interactions with F468 (of PH1), its hydrogen bond swapped from S501 (of the adjacent TM6) (in **Figure 1B**, left) to S436 (of TM5) and it could form an extra hydrogen bond through a water molecule with P461 (of PH1) (in **Figure 1B**, right). At the HC-030031 binding site, PS moved deeper into the cavity from its initial docking site, losing the initial interactions while forming a new water-mediated hydrogen bond with D412 (of the TM4-TM5 linker). This suggests that PS is too small for this site and that the A-967079 binding site is the preferred site for PS. Taken together, both PS and PSMME can bind to *Drosophila* TrpA1, according to the *in silico* studies.

### DSS-treatment causes microbial dysbiosis in *Drosophila* larvae and induces activation of Relish-mediated inflammatory gene expression

To study the effect of stilbenoid compounds *in vivo*, we wanted to induce activation of the NF-κB-mediated inflammatory Imd/Relish pathway in the *Drosophila* intestine, without direct interference of the signaling mediators of the pathway, or by bacterial infection. In both murine and *Drosophila* models, oral administration with DSS has been used to chemically induce intestinal inflammation (29–31). DSS has been shown to disrupt the epithelia barrier and the intestinal homeostasis, inducing intestinal inflammation (31–35).

**Figure 2:**
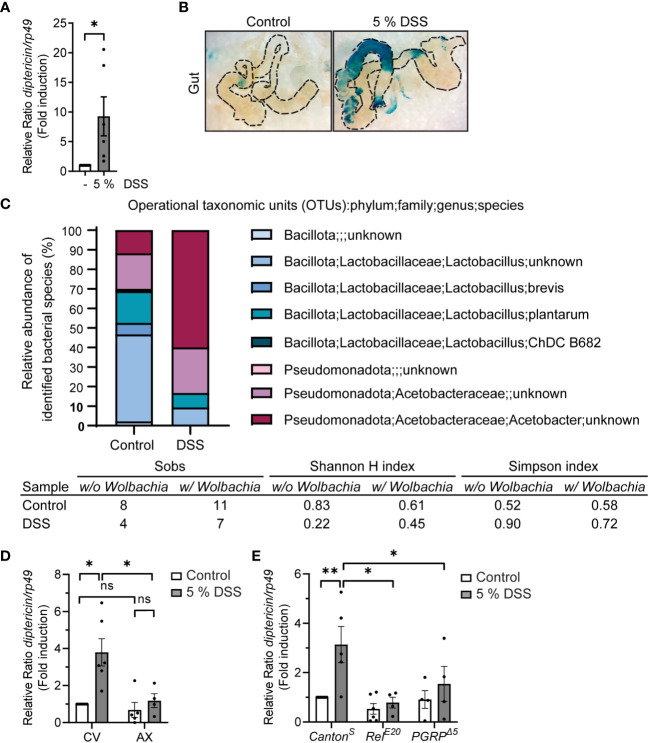
DSS-feeding induces local inflammation and causes microbial dysbiosis. **(A)** 3^rd^ instar larvae of wild-type *Canton^S^* were fed with indicated concentrations of DSS for 3 h, with 1 h recovery. Relish activation was studied by analyzing the expression of *diptericin* with qPCR (shown as ΔΔCt). Error bars indicate SEM from 6 independent experimental repeats. **(B)** Dissected larval guts from *diptericin-lacZ* stained for β-galactosidase activity after 3 hours with indicated concentrations of DSS feeding. The images are representatives of 3 independent experimental repeats. **(C)** Bacterial 16S rRNA metagenomics analysis of the 1V-3V region in *Canton^S^* control and DSS fed flies with 10% of DSS for 3 hours. Colors indicate identified operational taxonomic units (OTUs). The intestinal bacterial diversity of control and DSS treated wild-type *Canton^S^* larvae with and without *Wolbachia* was analysed by calculating the Simpson index, the Shannon-wiener H index, and the total number of observed families (Sobs). **(D)** 3^rd^ instar larvae of conventionally (CV) or axenically reared (AX) wild-type *Canton^S^* flies were fed with 5% DSS for 3 h with 1 h recovery. Relish activation was studied by analyzing the expression of *diptericin* with qPCR (shown as ΔΔCt). Error bars indicate SEM from 4 independent experimental repeats. **(E)** 3^rd^ instar larvae of wild-type *Canton^S^*, LOF *Rel^E20^* flies and *PGRP^Δ5^-*receptor mutant flies were fed with 5% DSS for 3 h with 1 h recovery. Relish activation was studied by analyzing the expression of *diptericin* with qPCR (shown as ΔΔCt). Error bars indicate SEM from more than 4 independent experimental repeats. Statistical significance was calculated using Student’s t-test **(A)** or one-way ANOVA **(D, E)** on non-normalized -ΔCt-values, ns nonsignificant, * p < 0.05, ** p < 0.01.

To assess the NF-κB-mediated inflammatory response induced by DSS in *Drosophila*, we fed foraging 3^rd^ instar larvae with fly food supplemented with DSS, whereafter, we used qPCR-based analysis to detect Relish-specific AMP target genes. We noticed that 5% w/v of 40 kDa DSS induced an increased gene expression of the NF-κB Relish target gene *diptericin* compared to control fed flies (**Figure 2A**), also in the absence of pathogenic infection. As Relish is involved in protecting the epithelial borders in the intestine from local insults, and thus maintains tissue homeostasis *in situ* also during non-pathogenic inflammation-inducing conditions (22, 36, 37), we wanted to specifically investigate if the local immune response in the gut is activated in response to DSS. As expected, we were able to detect local activation of the immune response in the *Drosophila* intestine by performing X-Gal staining on the dissected gut of *diptericin-lacZ* reporter flies fed with DSS (**Figure 2B**).

In addition, we analyzed if DSS treatment affects the intestinal microbiome in the fly. For this purpose, we performed 16S rRNA sequencing on control and DSS-fed larvae. The *Drosophila* gut harbors relatively few bacterial species, usually belonging to the families *Enterococcaceae* and *Lactobacillaceae* from the phylum *Bacillota*, and to the families *Acetobacteraceae* and *Enterobacteriaceae* from the phylum *Pseudomonadota* (38–40). We found the bacterial composition to be changed in DSS-treated larvae compared to control larvae (**Figure 2C**). The treatment with DSS leads to a decrease in the proportion of *Bacillota* to *Pseudomonadota* (**Figure 2C**, **Supplementary Figure S1** and **Table S1**), which is a marker for microbial instability and is associated with chronic inflammatory diseases also in humans (41, 42). Concomitantly, the Simpson index indicates a higher dominance and lower biodiversity in DSS treated larvae compared to control treated. Further supporting this notion, both the total number of observed families (Sobs) and the Shannon-wiener H index decreases in DSS treated larvae, indicating a decline in biodiversity (**Figure 2C**).

To assess if the fly commensal microbiome, alas dysbiotic, affects Relish activation upon DSS treatment, we reared flies under axenic conditions before treating them with DSS. Interestingly, DSS did not induce Relish activation in germ-free flies compared to their conventionally reared counterparts (**Figure 2D**). Similarly, the inducibility of *diptericin* is impaired in flies lacking the pattern-recognizing receptor (PRR) PGRP-LC (**Figure 2E**). This indicates that DSS-induced *diptericin* expression is not driven by disruption of the epithelial barrier, but rather mediated by receptor activation in response to a dysbiotic microbiome. In addition, this suggests that DSS-induced Relish target gene expression is mediated *via* activation of the Imd/Relish pathway. Finally, to assess if the DSS-induced expression of AMPs is indeed mediated by the NF-κB Relish, we used loss-of-function (LOF) mutants of Relish and confirmed that the inducibility of *diptericin* expression was Relish-dependent (**Figure 2E**). Taken together, DSS-treatment induces activation of Relish in flies and can be used to induce a modest inflammation in *Drosophila*.

### Stilbenoid compounds reduce inflammatory gene expression

**Figure 3:**
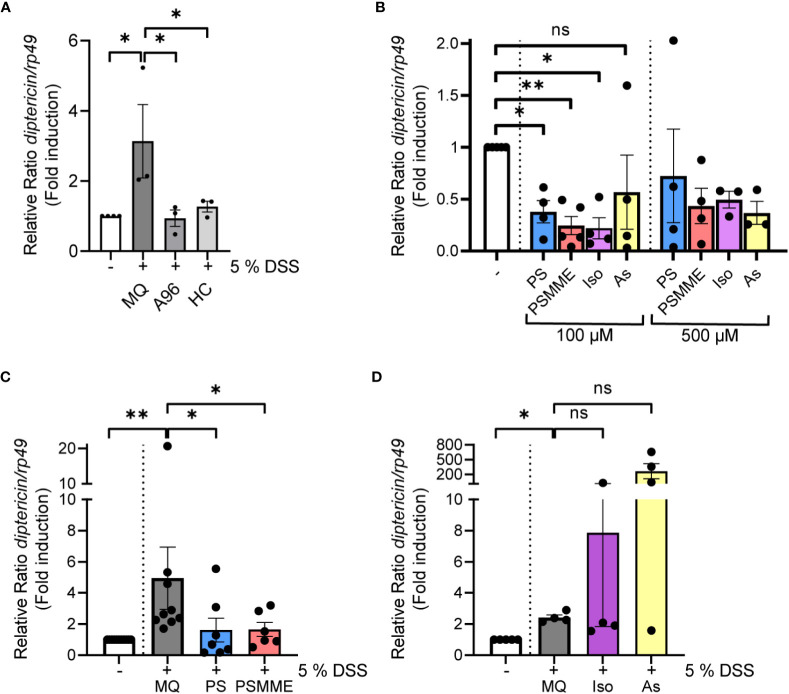
The anti-inflammatory properties of stilbenoid-compounds. **(A)** 3^rd^ instar larvae of wild-type *Canton^S^* were fed with 200 µM of TrpA1 antagonists A-967079 and HC-030031 or MQ for 24 hours after 3 hours DSS feeding. Relish activation was studied by analyzing the expression of *diptericin* with qPCR (shown as ΔΔCt). Error bars indicate SEM from 3 independent experimental repeats. **(B)** 3^rd^ instar larvae of wild-type *Canton^S^* were fed with indicated concentrations of stilbenoids PS, PSMME, isorhapontin and astringin for 24 hours. Relish activation was studied by analyzing the expression of *diptericin* with qPCR (shown as ΔΔCt). Error bars indicate SEM from more than 3 independent experimental repeats. **(C, D)** 3^rd^ instar larvae of wild-type *Canton^S^* were fed with 100 µM of stilbenoids PS **(C)**, PSMME **(C)**, isorhapontin **(D)** and astringin **(D)** or MQ for 24 hours after 3 hours DSS feeding. Relish activation was studied by analyzing the expression of *diptericin* with qPCR (shown as ΔΔCt). Error bars indicate SEM from more than 3 independent experimental repeats. Statistical significance was calculated using one-way ANOVA on non-normalized -ΔCt-values, ns nonsignificant, * p < 0.05, ** p < 0.01.

Taking advantage of the model of DSS-induced dysbiosis and inflammation, we wanted to investigate the anti-inflammatory properties of stilbenoid compounds in flies. To relate our modelling studies of stilbenes and the reference TrpA1 antagonists with the experimental data, we first determined the anti-inflammatory properties of the reference compounds A-967079 and HC-030031 in DSS-treated flies. We fed the DSS-treated flies with A-967079 and HC-030031, and both TrpA1-inhibiting drugs alleviated DSS-induced inflammation 24 hours post DSS-treatment (**Figure 3A**), suggesting that the stilbenoid compounds may also exert their anti-inflammatory properties by inhibiting the TrpA1 ion channel also in *Drosophila*.

Before assessing the anti-inflammatory properties of the stilbenoids, we first fed flies with the stilbenoids to investigate if the treatment alone activates NF-κB in *Drosophila*. We analyzed four different stilbenoid compounds, the compounds modelled together with *Drosophila* TrpA1 pinosylvin (PS) and pinosylvin monomethyl ether (PSMME), as well as the stilbenoid glucosides isorhapontin (4,5’-dihydroxy-3-methoxy-3’-glucopyranosylstilbene) and astringin (3,4,3’,5’-tetrahydroxystilbene 3’-glucoside). When used at a concentration of 100 µM, none of the tested stilbenoids induced inflammation after 24 hours of feeding. On the contrary, treatment with 100 µM PS, PSMME and isorhapontin reduced basal Relish target gene expression (**Figure 3B**). Astringin, on the other hand, did not seem to influence basal Relish activity (**Figure 3B**). Similar results were obtained when using a higher, 500 µM concentration of PSMME, isorhapontin and astringin (**Figure 3B**). However, a higher concentration of PS resulted in an adverse spontaneous increase of Relish target gene expression (**Figure 3B**).

To assess the anti-inflammatory effect of stilbenes, we fed larvae with DSS for 3 hours, after which they were allowed to feed on control food or food supplemented with stilbenoids for 24 hours. While DSS-treated flies still expressed *diptericin* 24 hours post DSS-treatment in control conditions, PS and PSMME, were able to alleviate the DSS-induced inflammation (**Figure 3C**). However, isorhapontin had no alleviating effect on DSS-induced inflammation and astringin seemed to have an opposite effect (**Figure 3D**). As both isorhapontin and astringin were unable to reduce DSS-induced inflammation the compounds were excluded from further experiments.

### Stilbenoid compounds depend on TrpA1 for their anti-inflammatory activity

**Figure 4:**
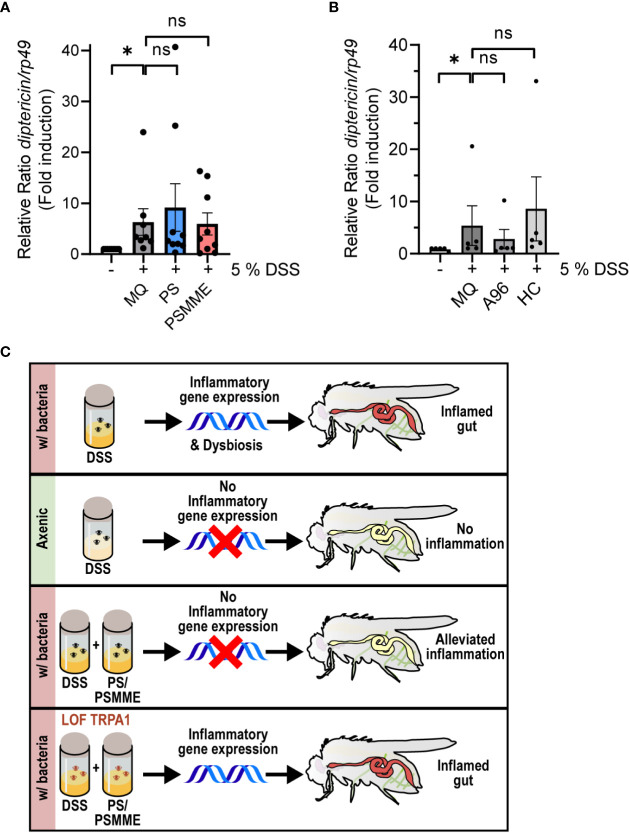
TrpA1-dependency of the stilbenoid compound anti-inflammatory activity. 3^rd^ instar larvae of LOF mutant *TrpA1^1^* were fed with 100 µM of stilbenoids PS and PSMME **(A)** or 200 µM of TrpA1 antagonists A-967079 and HC-030031 **(B)** or MQ for 24 hours after 3 hours DSS feeding. Relish activation was studied by analyzing the expression of *diptericin* with qPCR. Error bars indicate SEM from more than 5 independent experimental repeats (shown as ΔΔCt). Statistical significance was calculated using one-way ANOVA on non-normalized -ΔCt-values, ns nonsignificant, * p < 0.05. **(C)** Schematic summary of results showing that inhibition of the TrpA1 channel by stilbenoid-treatment reduces inflammation induced by Relish target gene expression.

To assess if the anti-inflammatory effect of PS and PSMME is indeed mediated *via* the TrpA1 channel *in vivo*, we investigated the ability of the stilbenoids to alleviate inflammation in TrpA1 LOF *TrpA1^1^*-mutant flies. We first ensured that the larvae of TrpA1 LOF flies responded to DSS-treatment by inducing expression of *diptericin* similarly as wildtype flies (**Supplementary Figure S2**). At the same time this indicates that the DSS-induced Relish activation is TrpA1-independent. When we next treated the DSS-fed *TrpA1*-mutant larvae with stilbenoid compounds, PS and PSMME lost their anti-inflammatory properties observed in control larvae (**Figure 4A**). Finally, the DSS-induced *diptericin* expression could not be alleviated by feeding TrpA1 LOF flies with the known antagonists of mammalian TrpA1, A-967079 and HC-030031 (**Figure 4B**), hence, strengthening the functional role of TrpA1 in the immune response during DSS-induced intestinal inflammation (**Figure 4C**).

## Discussion

When intestinal cellular and microbiome homeostasis is disturbed, as in inflammatory bowels disease (IBD) patients, activation of the transcription factor NF-κB in the epithelium is markedly induced, further promoting intestinal inflammation (43, 44). In this study, we demonstrate that feeding *Drosophila* larvae with DSS leads to an intestinal inflammatory response mediated *via* the NF-κB transcription factor Relish. Interestingly, our results indicate that DSS-induced Relish activation is not prompted by the damage in the epithelial barrier, but instead through activation of inflammatory receptors as a response to the emerging microbial instability. Therefore, the DSS-induced inflammatory response caused by a microbial dysbiosis of commensal bacteria is modest in comparison to immune responses caused by pathogenic infections. While the activation of Relish seems to be TrpA1-independent, feeding flies with DSS has been shown to increase ROS levels (34), which in turn, is suggested to activate the TrpA1 ion channels expressed in the epithelial cells along the *Drosophila* midgut (13, 14). Interestingly, TrpA1 is upregulated in IBD patients and its activation is required to alleviate the expression of several proinflammatory neuropeptides, cytokines and chemokines (45). Here, we show that although TrpA1 is not required for DSS-induced Relish activation, there is a crosstalk between TrpA1 and Relish, as the DSS-induced Relish activation can be modulated pharmacologically with stilbenoid compounds PS and PSMME that target TrpA1.

In this study, we computationally modelled the putative binding interactions of PS and PSMME at *Drosophila* TrpA1. We specifically focused on investigating the binding sites reported for known human TrpA1-antagonists A-967079 and HC-030031. Previous studies have stated that the predicted A-967079 binding site in mammalian TrpA1 consists of different residues than the corresponding pocket in the *Drosophila* TrpA1, thus rendering *Drosophila* TrpA1 insensitive to A-967079 (24, 46, 47). The docking study and the MD analysis of the putative binding poses of the stilbenes showed that PS can form favorable polar and hydrophobic interactions with the target at both studied sites. While PS showed comparable predicted affinities to both antagonist binding sites, its size and interactions suggest that it may favor the A-967079 binding pocket. A-967079 however, did not find a stable binding site at *Drosophila* TrpA1, consistent with the previous observation that *Drosophila* TrpA1 is insensitive to A-967079. However, when analyzing *in vivo*, A-967079 did have anti-inflammatory properties, indicating that A-967079 may bind to TrpA1 sufficiently *in vivo*. While the stilbenoids resveratrol, PS and PSMME have been previously shown to have anti-inflammatory properties *in vivo* (7, 8, 48), isorhapontin and astringin have not been studied *in vivo* before. We were not able to detect any significant anti-inflammatory effects with neither of these compounds. However, isorhapontin and astringin are both hydrophilic glucosides that may not diffuse through the lipophilic cell membrane to the lipid-surrounded binding site at TrpA1, and would hence need to be metabolized to function properly (49).

Besides their direct interaction with the TrpA1-receptor, stilbenoid compounds also exhibit antimicrobial effects (50, 51), making them potential modulators of the bacteria composition in the intestinal lumen. This could explain why during basal conditions, some of the stilbenoid compounds were able to alleviate basal NF-κB activation. As the microbial dysbiosis caused by DSS seems to be the main reason for Relish activation, the antimicrobial activities of stilbenoids may alleviate the bacterial burden on the intestinal epithelia, which may contribute to the anti-inflammatory effect of PS and PSMME. To further address this, an analysis of the microbial structure in response to stilbenoid treatments would be informative and would elucidate the antimicrobial effects of stilbenoids during intestinal inflammation. In conclusion, both our molecular modelling and our experimental data indicate favorable interactions of the stilbenoid compounds with the *Drosophila* TrpA1, suggesting crosstalk between TrpA1 and NF-κB signaling in maintenance of intestinal immune homeostasis.

## Materials and methods

### Fly husbandry and strains

*Drosophila melanogaster* were maintained at 25°C with a 12 h light–dark cycle on Nutri-fly BF (Dutscher Scientific, Essex, UK). *Canton^S^* wildtype flies and *diptericin-lacZ* reporter line and balancer lines fly lines were kindly provided by Prof. Pascal Meier and Dr. François Leulier (52, 53). The *Drosophila* fly lines *TrpA1^1^* (#36342), *PGRP-LC^Δ5^* (#36323), *w:Rel^E20^* (stock #9457) were obtained from the Bloomington stock center.

### DSS treatment of *Drosophila* larvae

Early *Drosophila* 3^rd^ instar larvae were fed 40 kDa DSS (TdB Consultancy AB, Uppsala, Sweden) mixed in the fly food. 40 kDa DSS has been shown to induce the most severe colitis in mammalian model organism (54). For feeding with DSS 3-10 larvae were placed in 2 ml collection tubes with food (1-2 ml) containing DSS and sealed with pieces of *Drosophila* plugs (Genesee Scientific, California, USA). For qPCR experiments, more than 3 larvae were fed for 3 h with indicated DSS concentrations (1%, 5% or 10% w/v) and allowed to recover for at least 1 h before freezing. As DSS has been shown to compromise qPCR results by interfering with the activity of reverse transcriptase during cDNA synthesis and the polymerase during qPCR (55), we decided to use a one-hour recovery post DSS-treatment in all qPCR-experiments. For sequencing of the 16S rRNA gene larvae were fed with 10% w/v DSS for 3 h in room temperature with a 2 h recovery.

### Stilbenoid compounds

PS and PSMME were isolated from a mixture of Norway spruce and Scot pine knotwood using a batch reactor. In the first step of isolation, the wood materials were boiled in ethanol for about two hours. After cooling, the ethanol solution was filtrated and then condensed by distillation (56). Further, the ethanol extract was dissolved in Toluene and stirred in a dark place at room temperature to remove the polymers that could interfere with the purification process. After two days, it was filtrated, and the solid residue was returned to the flask. This procedure was repeated two more times. The final filtrate was evaporated with a rotary evaporator at 40℃. The extract was subjected to a normal phase column chromatography (Silica gel 60, 0.040–0.063 mm, Merck, Darmstadt, Germany) and eluted with petroleum ether (peth) and ethyl acetate (EtOAc) as a solvent system. PSMME was separated in a proportion of 80:20 (peth/EtOAc). The remaining obtained fractions contained PS, resin acids and hydroxymatairesinol (HMR) were subjected to the second normal phase column and eluted with 100% chloroform (CHCl3). The procedure continued by using a gradient of CHCl3/MeOH (98:2) and the fractions were collected based on TLC (Silica gel 60 F254, Merck, Darmstadt, Germany) profile. All the organic solvents in analytical grade (99.9%) were purchased from Sigma-Aldrich (St. Louis, MO, USA). The purity of the fractions containing PSMME and PS was analyzed by GC-MS after silylation (**Supplementary Figure S3, S4**). The purity was determined to be >95% (**Supplementary Figure S5, S6**). The silylation reagents hexamethyldisiloxane (HMDS) and trimethylchlorosilane (TMSCl) were purchased from Sigma-Aldrich (St. Louis, MO, USA) and pyridine was obtained from VWR (Fontenay-sous-Bois, France). The GC-MS instrument was Agilent 5975C TAD series GC/MSD system (Stevens Creek, Santa Clara, CA, USA). The stilbenoid glucosides (astringin and isorhapontin) were extracted from fresh inner bark of Norway spruce by acetone and further purified by column chromatography over 95% and analyzed by GC-MS and NMR described in detail in our previously reported method (57).

### Stilbenoid and TrpA1 antagonist treatment of *Drosophila* larvae

Similarly as with DSS feeding, 3-10 larvae were fed 24 h with indicated concentrations (100 µM or 500 µM) of stilbenoid compounds PS, PSMME, isorhapontin and astringin, or 200 µM of TrpA1 antagonists A-967079 (Sigma-Aldrich) and HC-030031 (Sigma-Aldrich) mixed in 1-2 ml fly food. For experiments with DSS treatment, larvae were moved from DSS-containing food to new tubes with stilbene/antagonist-containing food.

### Quantitative real-time-PCR

*Drosophila* larvae were homogenized using QIAshredder (QIAGEN) and total RNA was extracted with RNeasy Mini Kit (QIAGEN) and cDNA was synthesized with SensiFast cDNA synthesis kit (Bioline, London, UK) according to the manufacturers’ protocols. qPCR was performed using SensiFast SYBR Hi-ROX qPCR kit (Bioline). *rp49* was used as a housekeeping gene for ΔΔCt calculations. The following gene-specific primers were used to amplify cDNA: *diptericin* (5’-ACCGCAGTACCCACTCAATC-3’, 5’-ACTTTCCAGCTCGGTTCTGA-3’), *rp49* (5’-GACGCTTCAAGGGACAGTATCTG-3’, 5’-AAACGCGGTTCTGCATGAG-3’).

### X-gal staining of *Drosophila* larvae

3^rd^ instar fly larvae were dissected in PBS and fixed for 15 minutes with PBS containing 0.4% glutaraldehyde (Sigma-Aldrich) and 1 mM MgCl_2_ (Sigma-Aldrich). The samples were washed with PBS and incubated with a freshly prepared staining solution containing 5 mg/ml X-gal (5-Bromo-4-chloro-3-indolyl-β-D-galactopyranoside), 5 mM potassium ferrocyanide trihydrate (Sigma-Aldrich), 5 mM potassium ferrocyanide crystalline (Sigma-Aldrich) and 2 mM MgCl_2_ in PBS at 37°C. After washing with PBS, the samples were mounted using Mowiol (Sigma) and imaged with brightfield microscopy (Leica, Wetzlar, Germany).

### Sequencing of the 16S rRNA gene

Genomic DNA was isolated from 40 3^rd^ instar larvae using a modified protocol for the QIAamp DNA mini kit (QIAGEN) (58). Larvae were surface sterilized by vortexing them twice in 2% active hypochlorite and sterile H_2_O. The efficiency of the washes was confirmed by 16S PCR of water from the last wash step. Larvae were homogenized in lysis buffer containing 20 mM Tris, pH 8.0, 2 mM EDTA, 1.2% Triton X-100 and 20 mg/ml lysozyme and incubated 90 min at 37°C. 200 µl AL buffer (QIAamp DNA mini kit) with 20 µl proteinase K were added and the lysate was incubated 90 min at 56°C. Subsequent extraction was performed according to manufacturer’s protocol. Amplification and Illumina MiSeq sequencing of the V1-V3 region of the 16S rRNA gene, as well as selection of operational taxonomic units (OTUs) and taxonomy assignment of OTUs was done using Eurofins Genomics InView Microbiome Profiling 3.0 service. The data is deposited at NCBI, BioProject ID: PRJNA1005106. The proportion of *Wolbachia* species have been omitted from the bar graph in **Figure 2C** for easier comparison of bacterial species residing in the gut lumen. However, Wolbachia is included in **Figure 2C**, **Supplementary Figure S1** and **Supplementary Table S1**.

### Computational protein modelling

The fruit fly (*Drosophila melanogaster*) TrpA1 (dTrpA1) sequence was obtained from the UniProt knowledgebase (UniProtKB - Q7Z020). A Basic Local Alignment Search Tool (BLAST) (59) search for the sequence was run to find a suitable template in the Protein Data Bank (PDB) (60) for comparative modelling of its three-dimensional (3D) structure. Among protein structures with similar E-values (0.0) and Sequence Identity (35.81%), the human TrpA1 crystal structure (PDB ID: 6V9V) (61) was selected due to the highest resolution (2.60 Å) and the greatest sequence coverage (residues 1 – 1119). The 3D structure model of dTrpA1 was generated using Modeller (v. 9.24) (62). The modelling alignment was created with Clustal Omega (63) and manually curated. Out of the 20 generated alternative models, the one with the best Discrete Optimized Protein Energy (DOPE) score (64) was selected. The model was further evaluated by superimposition on the template structure using PyMOL (The PyMOL Molecular Graphics System, Version 4.6, Schrödinger, LLC) and the stereochemical quality of the model was verified with MolProbity (65).

### Computational docking studies

The structures of natural stilbenes and the reference compounds were prepared using the LigPrep module of Maestro (Release 2020-2: Schrödinger, LLC, New York, NY, 2020) software and the Protein Preparation Wizard of Maestro (66) was used to minimize the dTrpA1 model using the OPLS3e force field (67) and the RMSD of 0.3 Å for heavy atoms as the convergence criteria. Two docking sites were defined based on the previously reported TrpA1 antagonist binding pockets (28) using the Receptor Grid Generation tool of Maestro: (i) A-967079 binding pocket around the amino acids V431, L432, F435, F468 and M471; and (ii) HC-030031 binding site around W268, Q413, Q538, H542 and A530. The stilbene compounds were docked at these alternative sites with the GLIDE docking tool (68–70) of Maestro, using the extra precision (XP) mode with flexible ligand sampling. A maximum of five poses per ligand were ranked based on the Glide XP docking score (XP Gscore) value. To allow for more accurate evaluation of the predicted docking poses, binding free energy calculations with the Prime/MM-GBSA module of Maestro (71) were carried out for the best-docked pose (according to the XP Gscore and the observed binding interactions) of each compound using the VSGB 2.0 solvation model (72) and the OPLS3e force field (67) and allowing the residues within 5 Å from the ligand to move.

### Molecular dynamics simulation analysis

The simulation systems (consisting of the membrane embedded dTrpA1 and the receptor-bound ligand in explicit solvent) were created with the System Builder tool of the Desmond module (Schrödinger Release 2020-2: Desmond Molecular Dynamics System, D. E. Shaw Research, New York, NY, USA, 2020. Maestro-Desmond Interoperability Tools, Schrödinger, New York, NY, USA, 2020) (73) using TIP3P water (74) as the solvent model and POPC (1-palmitoyl-2-oleoyl-sn-glycero-3-phosphocholine) as the membrane model. The systems were neutralized by adding Na^+^ counter ions. After the system relaxation, the production simulations were run for 100 ns at constant temperature (300 K) and pressure (1.01325 bar) according to our previously reported simulation protocol (75).

### Statistical analysis

Results from qPCR were analyzed by ordinary one-way ANOVA and two-tailed Student’s t-test on the non-normalized -ΔCt values, the graphs depict relative fold induction of the target gene compared to a normalized sample (ΔΔCt). Statistical analyses were performed using GraphPad Prism version 9.5.0 for Windows (GraphPad Software, San Diego, California, USA). In figures, ns stands for p> 0.05, * p < 0.05, ** p < 0.01, *** p < 0.001, **** p < 0.0001. Error bars in figures specify SEM from the indicated number of independent experimental repeats with more than 3 larvae per treatment. Experiments were performed indicated number of times (n ≥ 3) and statistics were calculated for each individual experiment. For analysis of 16S rRNA sequencing data, Shannon-wiener H index was calculated according to H = -Σpi*ln(pi) and Simpson index was calculated according to D = Σni(ni-1)/N(N-1). In addition, the total number of observed families (Sobs) was calculated.

## Data availability statement

The data presented in the study are deposited in the NCBI repository, accession number PRJNA1005106, https://www.ncbi.nlm.nih.gov/sra/PRJNA1005106.

## Ethics statement

The studies involving transgenic flies and genetically modified material were reviewed and approved by the Finnish Board for Gene Technology (Reg. Nr. 007/S/2021).

## Author contributions

AA: Conceptualization, Formal Analysis, Funding acquisition, Investigation, Methodology, Writing – original draft, Writing – review & editing. AS: Investigation, Writing – original draft. FL: Investigation, Writing – review & editing. CK: Investigation, Writing – review & editing. EH: Investigation, Writing – review & editing. PM: Methodology, Supervision, Writing – review & editing. OS: Conceptualization, Supervision, Writing – review & editing. PE: Conceptualization, Supervision, Writing – original draft, Writing – review & editing. AM: Conceptualization, Funding acquisition, Methodology, Project administration, Resources, Supervision, Writing – original draft, Writing – review & editing.

## Supplementary material

Supplementary Figure 1 Bacterial 16S rRNA metagenomics analysis of the 1V-3V region, including and excluding Wolbachia.

Supplementary Figure 2 Comparison of *Diptericin* expression in wildtype *Canton^S^* and TRPA1 LOF flies in response to DSS treatment.
