# Supplementary material for: Stilbenoid compounds inhibit NF-κB-mediated inflammatory responses in the Drosophila intestine
Source: Front Immunol. 2023 Sep 22;14:1253805. doi: 10.3389/fimmu.2023.1253805 (PMC10556681; doi:10.3389/fimmu.2023.1253805)
Supplement: Supplementary file 1 [file DataSheet_1.pdf]

Supplementary Material (Aalto et al)

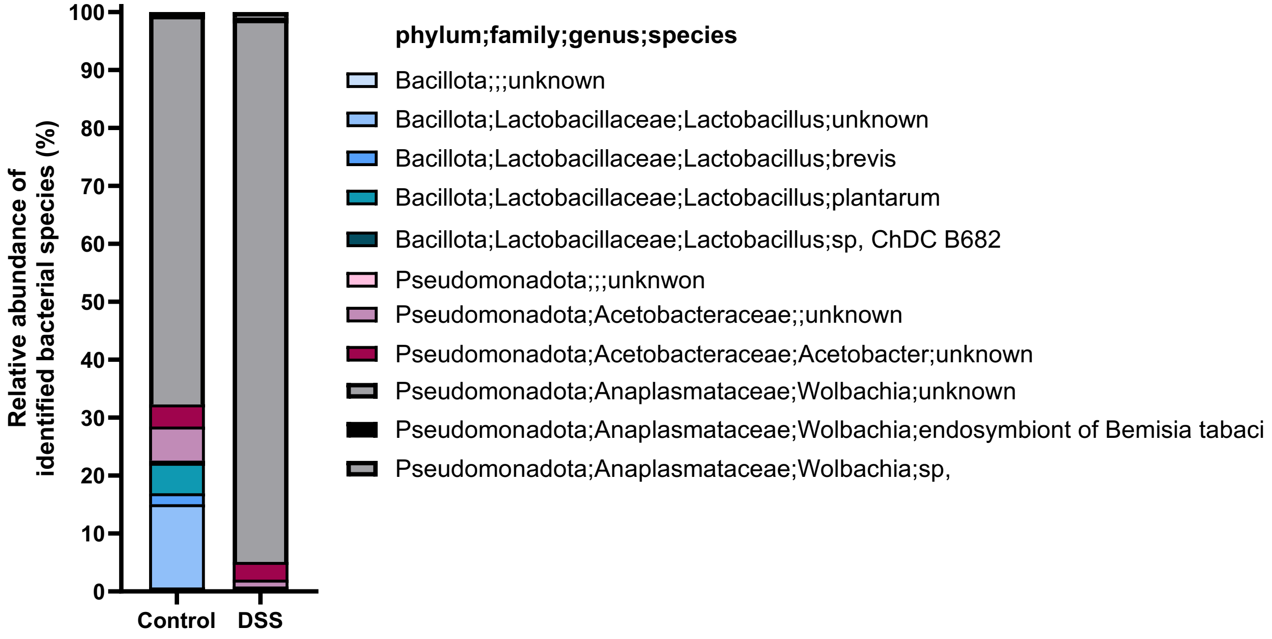

Supplementary Figure 1. Bacterial 16S rRNA metagenomics analysis of the 1V-3V region in *Canton^S* control and DSS fed flies with 10 % of DSS for 3 hours. Colours indicate identified operational taxonomic units (OTUs).

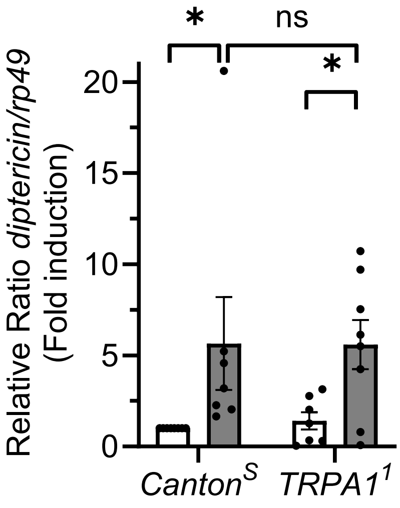

Supplementary Figure 2. 3rd instar larvae of wild-type *Canton^S* and LOF mutant *TrpA1^1* were fed with DSS for 3 hours, with 24 hour recovery. Relish activation was studied by analysing the expression of *diptericin* with qPCR. Error bars indicate SEM from more than 5 independent experimental repeats. Statistical significance was calculated using one-way ANOVA on non-normalised -ΔCt-values, ns nonsignificant, * $p < 0.05$.

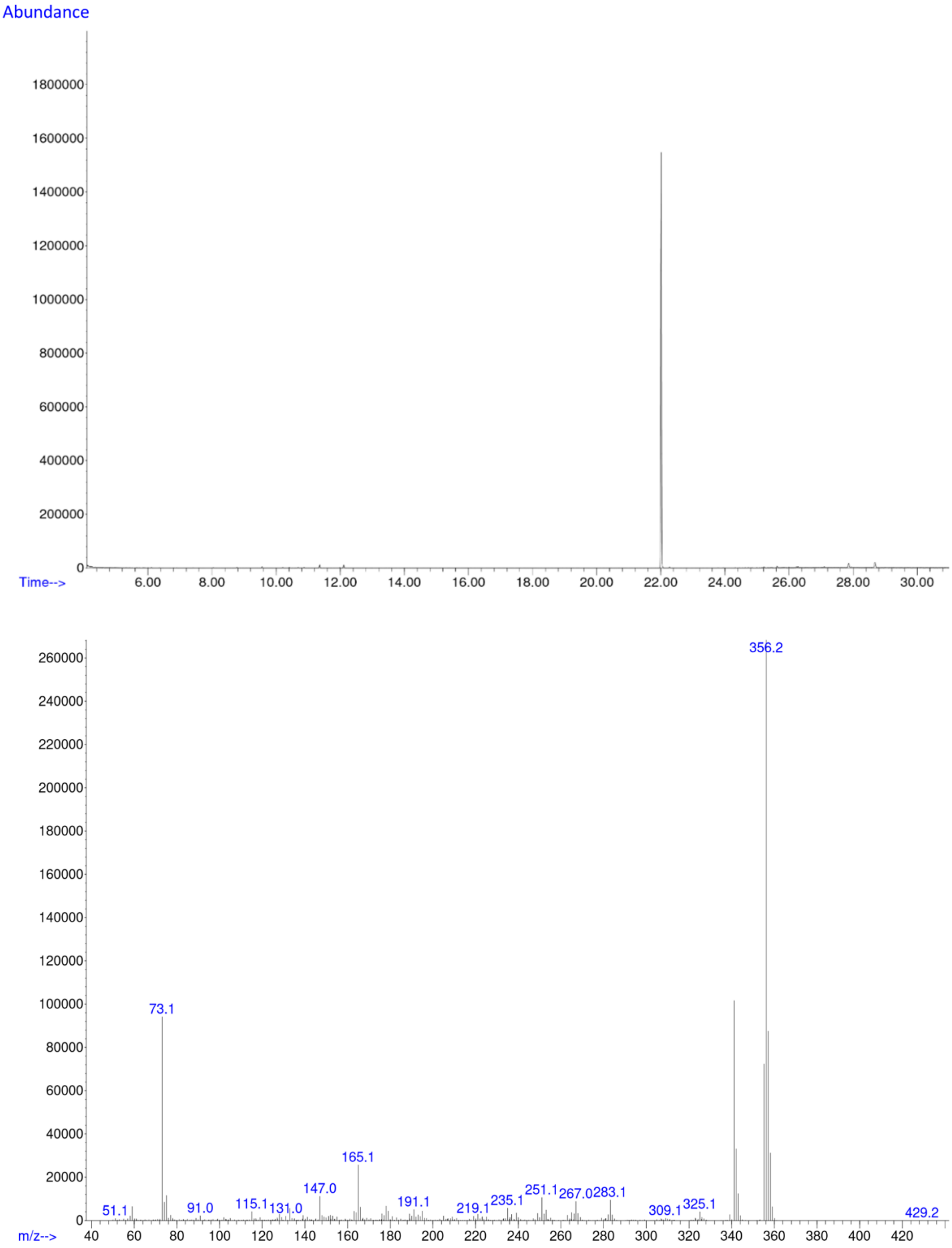

Supplementary Figure 3. GC-MS chromatogram of silylated PS.

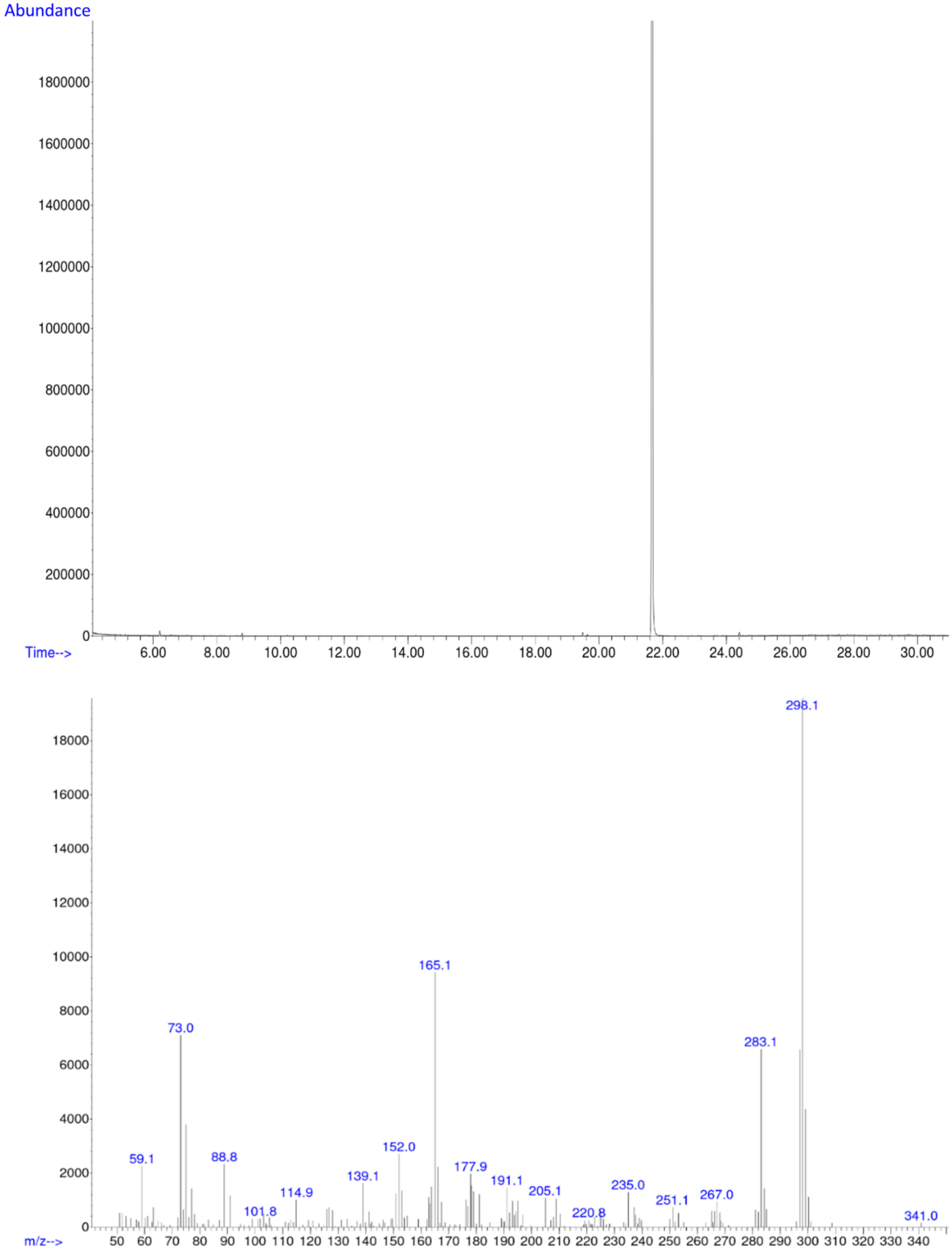

Supplementary Figure 4. GC-MS chromatogram of silylated PSMME.

Area Percent Report

Data Path : C:\msdchem\2\data\Ati\Natural compounds\
Data File : Ps-Col-CHCL3-MeOH.D
Acq On : 3 May 2019 13:02
Operator : Ati
Sample :
Misc :
ALS Vial : 15 Sample Multiplier: 1

Integration Parameters: autoint1.e
Integrator: ChemStation

Method : C:\MSDCHEM\2\METHODS\default.m
Title :

Signal : TIC: Ps-Col-CHCL3-MeOH.D\data.ms

peak #	R.T. min	first scan	max scan	last scan	PK TY	peak height	corr. area	corr. % max.	% of total
1	22.015	2441	2448	2460	BB	1464960	22486801	100.00%	100.000%

Sum of corrected areas: 22486801

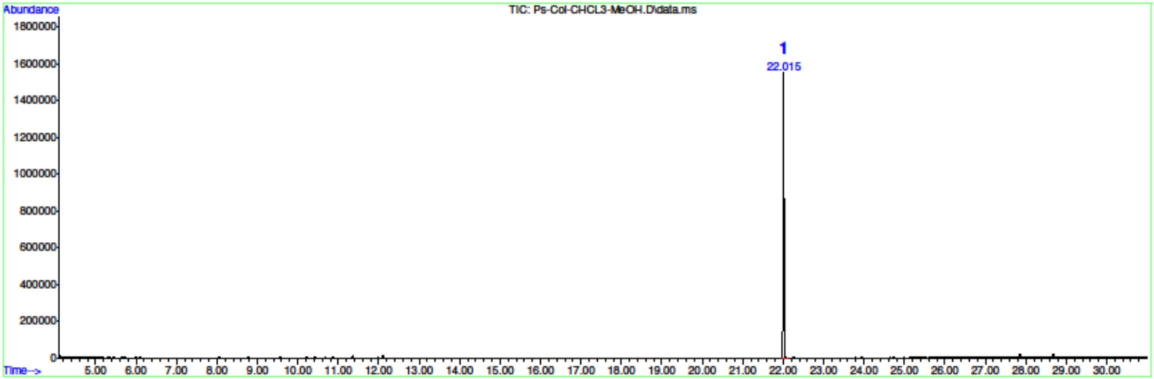

Supplementary Figure 5. Area percent report for PS.

Area Percent Report

Data Path : C:\msdchem\2\data\Ati\Natural compounds\
Data File : PsMME-Pure-no silylation.D
Acq On : 29 Mar 2019 12:18
Operator : Ati
Sample :
Misc :
ALS Vial : 19 Sample Multiplier: 1

Integration Parameters: autoint1.e
Integrator: ChemStation

Method : C:\MSDCHEM\2\METHODS\default.m
Title :

Signal : EIC TIC: PsMME-Pure-no silylation.D\data.ms

peak #	R.T. min	first scan	max scan	last scan	PK TY	peak height	corr. area	corr. % max.	% of total
1	21.656	2379	2399	2404	BV	181065	3187848	1.41%	1.393%
2	21.758	2404	2413	2466	VV	12083217	225643927	100.00%	98.607%

Sum of corrected areas: 228831775

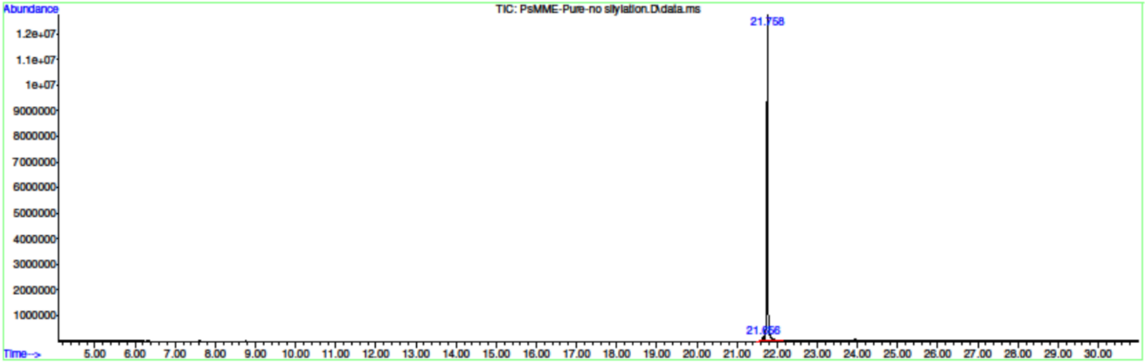

Supplementary Figure 6. Area percent report for PSMME.

Supplementary Table 1. Taxonomy table. The entries of the matrix are the estimated abundances of the respective taxonomic unit/sample combination of the bacterial 16S rRNA metagenomics analysis of the V1-V3 region in *Canton*^{S} control and DSS fed flies with 10 % of DSS for 3 hours. Colours indicate identified operational taxonomic units (OTUs).

Excluding Wolbachia	Absolute abundance	Relative abundance (%)	Absolute abundance	Relative abundance (%)
#OTU ID	Control_CantonS.V1V3	Control_CantonS.V1V3	DSS_CantonS.V1V3	DSS_CantonS.V1V3
k__Bacteria;p__Bacillota;c__Bacilli;o__Lactobacillales;f__;g__;s__	355	2,2	0	0,0
k__Bacteria;p__Bacillota;c__Bacilli;o__Lactobacillales;f__Lactobacillaceae;g__Lactobacillus;s__	7266	44,5	481	9,3
k__Bacteria;p__Bacillota;c__Bacilli;o__Lactobacillales;f__Lactobacillaceae;g__Lactobacillus;s__brevis	960	5,9	0	0,0
k__Bacteria;p__Bacillota;c__Bacilli;o__Lactobacillales;f__Lactobacillaceae;g__Lactobacillus;s__plantarum	2654	16,3	380	7,4
k__Bacteria;p__Bacillota;c__Bacilli;o__Lactobacillales;f__Lactobacillaceae;g__Lactobacillus;s__sp. ChDC B682	73	0,4	0	0,0
k__Bacteria;p__Pseudomonadota;c__;o__;f__;g__;s__	118	0,7	0	0,0
k__Bacteria;p__Pseudomonadota;c__AlphaPseudomonadota;o__Rhodospirillales;f__Acetobacteraceae;g__;s__	2979	18,2	1201	23,3
k__Bacteria;p__Pseudomonadota;c__AlphaPseudomonadota;o__Rhodospirillales;f__Acetobacteraceae;g__Acetobacter;s__	1927	11,8	3094	60,0
Total	16332	100	5156	100

Including Wolbachia	Absolute abundance	Relative abundance (%)	Absolute abundance	Relative abundance (%)
#OTU ID	Control_CantonS.V1V3	Control_CantonS.V1V3	DSS_CantonS.V1V3	DSS_CantonS.V1V3
k__Bacteria;p__Bacillota;c__Bacilli;o__Lactobacillales;f__;g__;s__	355	0,7	0	0,0
k__Bacteria;p__Bacillota;c__Bacilli;o__Lactobacillales;f__Lactobacillaceae;g__Lactobacillus;s__	7266	14,4	481	0,5
k__Bacteria;p__Bacillota;c__Bacilli;o__Lactobacillales;f__Lactobacillaceae;g__Lactobacillus;s__brevis	960	1,9	0	0,0
k__Bacteria;p__Bacillota;c__Bacilli;o__Lactobacillales;f__Lactobacillaceae;g__Lactobacillus;s__plantarum	2654	5,2	380	0,4
k__Bacteria;p__Bacillota;c__Bacilli;o__Lactobacillales;f__Lactobacillaceae;g__Lactobacillus;s__sp. ChDC B682	73	0,1	0	0,0
k__Bacteria;p__Pseudomonadota;c__;o__;f__;g__;s__	118	0,2	0	0,0
k__Bacteria;p__Pseudomonadota;c__AlphaPseudomonadota;o__Rhodospirillales;f__Acetobacteraceae;g__;s__	2979	5,9	1201	1,2
k__Bacteria;p__Pseudomonadota;c__AlphaPseudomonadota;o__Rhodospirillales;f__Acetobacteraceae;g__Acetobacter;s__	1927	3,8	3094	3,1
k__Bacteria;p__Pseudomonadota;c__AlphaPseudomonadota;o__Rickettsiales;f__Anaplasmataceae;g__Wolbachia;s__	34045	67,3	94641	93,7
k__Bacteria;p__Pseudomonadota;c__AlphaPseudomonadota;o__Rickettsiales;f__Anaplasmataceae;g__Wolbachia;s__endosymbiont of Bemisia tabaci	103	0,2	208	0,2
k__Bacteria;p__Pseudomonadota;c__AlphaPseudomonadota;o__Rickettsiales;f__Anaplasmataceae;g__Wolbachia;s__sp.	136	0,3	976	1,0
Total	50616	100	100981	100
